# Caffeine Use among Active Duty Navy and Marine Corps Personnel

**DOI:** 10.3390/nu8100620

**Published:** 2016-10-09

**Authors:** Joseph J. Knapik, Daniel W. Trone, Susan McGraw, Ryan A. Steelman, Krista G. Austin, Harris R. Lieberman

**Affiliations:** 1 Military Nutrition Division, US Army Research Institute of Environmental Medicine, Natick, MA 01760, USA; susan.m.mcgraw6.civ@mail.mil (S.M.); krista.g.austin.ctr@mail.mil (K.G.A.); harris.r.lieberman.civ@mail.mil (H.R.L.); 2 US Army Public Health Center, Aberdeen Proving Ground, MD 21010, USA; ryan.a.steelman.ctr@mail.mil; 3 Oak Ridge Institute for Science and Education, Belcamp, MD 21017, USA; 4 Naval Health Research Center, San Diego, CA 92152, USA; daniel.w.trone.civ@mail.mil

**Keywords:** coffee, tea, cola, energy drink, alcohol, sleep, exercise, demographics, lifestyle characteristics

## Abstract

Data from the National Health and Nutrition Examination Survey (NHANES) indicate 89% of Americans regularly consume caffeine, but these data do not include military personnel. This cross-sectional study examined caffeine use in Navy and Marine Corps personnel, including prevalence, amount of daily consumption, and factors associated with use. A random sample of Navy and Marine Corps personnel was contacted and asked to complete a detailed questionnaire describing their use of caffeine-containing substances, in addition to their demographic, military, and lifestyle characteristics. A total of 1708 service members (SMs) completed the questionnaire. Overall, 87% reported using caffeinated beverages ≥1 time/week, with caffeine users consuming a mean ± standard error of 226 ± 5 mg/day (242 ± 7 mg/day for men, 183 ± 8 mg/day for women). The most commonly consumed caffeinated beverages (% users) were coffee (65%), colas (54%), teas (40%), and energy drinks (28%). Multivariable logistic regression modeling indicated that characteristics independently associated with caffeine use (≥1 time/week) included older age, white race/ethnicity, higher alcohol consumption, and participating in less resistance training. Prevalence of caffeine use in these SMs was similar to that reported in civilian investigations, but daily consumption (mg/day) was higher.

## 1. Introduction

Caffeine is a widely consumed psychoactive substance. Recent data from nationally representative samples indicate about 89% of American adults consume caffeinated products daily with virtually no difference between men and women in how frequently the products are used [1,2]. There has been some concern regarding the potential adverse health effects of caffeine, especially with regard to cardiovascular disease and diabetes mellitus risk. Acute caffeine consumption increases blood pressure and plasma lipids, and these effects led to speculation that chronic caffeine consumption would increase cardiovascular disease risk [3,4,5]; however, epidemiological studies have generally concluded that caffeine consumption is not associated with higher cardiovascular risk [6,7,8,9]. Short-term metabolic studies have suggested caffeine has adverse effects on glucose tolerance and whole-body glucose management [10,11] which could increase diabetes risk, but longer-term investigations found that coffee consumption was associated with a lower risk of Type 2 diabetes [7,12,13]. Other studies suggested there were positive effects of caffeinated products, including reduced liver dysfunction [14], slower age-related cognitive declines [15], improvement in some types of athletic performances [16], and reduced intensity of exercise-induced delayed onset muscle soreness [17]. A comprehensive review of the health effects of caffeine concluded that adult consumption ≤400 mg/day (6 mg/kg body weight for a 65 kg person) was not associated with adverse effects on cardiovascular health, bone status, male reproductive systems, adult behavior, or cancer risk, although it was recommended that women of reproductive age should consume <300 mg/day because of possible effects on fertility and conception [18].

Investigations that have obtained representative data on caffeine intake in Americans [1,2,19] have not provided information on United States (US) military service members (SMs). One previous study did report on caffeine use in Army personnel [20]. That investigation found that the prevalence of caffeine use was slightly lower than the general US population (82%), but among caffeine consumers, daily intake was considerably higher (347 mg/day). Factors associated with higher caffeine use included male sex, race/ethnicity other than black, and tobacco use.

The physical demands of the military, such as early morning physical training and limited sleep during training, operations, and deployments, may lead SMs to consume more caffeinated substances than the general population. The aim of this report was to examine the caffeine consumption of active-duty Navy and Marine Corps personnel and investigate characteristics associated with consumption. This is the second of a series of studies [20] designed to examine caffeine use in the military services.

## 2. Materials and Methods

This investigation was a cross-sectional survey of caffeine use among US active-duty Navy and Marine Corps personnel and was approved by the Naval Health Research Center’s (NHRC) institutional review board. Investigators requested information from the Defense Manpower Data Center (DMDC) on a random sample of 4000 Navy personnel (3000 men and 1000 women) and 6000 Marine Corps personnel (4500 men and 1500 women) currently on active duty and with at least 6 months of service as of February 2014 (10,000 personnel in total). Data obtained from DMDC included the SM’s name, branch of service, pay grade (rank), postal address, e-mail address, gender, age, marital status, and education level. National Change of Address records provided by the United States Postal Service were referenced to ensure the most up-to-date postal address was used. The random sample request to DMDC was based on previous experience with similar NHRC questionnaire investigations indicating an approximate 20% response rate from Navy and Marine Corps personnel [21] and statistical power considerations. SMs were eligible for inclusion in the study if they were on the list provided by DMDC and were on active duty in the Navy or Marine Corps.

### 2.1. Recruitment Procedures

Recruitment of participants in the random sample involved a maximum of six sequential contacts. The prospective participant was first sent an introductory postal letter including information about the purpose of the study, the investigators and their command affiliations, the sponsors, and the reason for conducting the study. The introductory letter provided the SM with a pre-incentive $10 gift card to nationally available businesses to encourage participation. The letter also included a description of the survey, a link to a secure website, and a subject identification number that could be used to access the survey and electronically sign the consent form. A follow-up email message after 10 days and postcard after three weeks were sent as reminders to those who did not initially complete the survey. If no response was received after sending the postcard, up to three additional email reminders were sent over three months, after which contact with the SM ended. Those who responded were sent a “Thank You” email message. All postal and on-line contacts stated that at any time the SM could decline participation and be removed from the contact list. Recruitment began in August 2014, and no further recruitment efforts were conducted or surveys accepted after December 2014.

### 2.2. Survey (Questionnaire) Description

The first section of the questionnaire was designed to characterize participants. Questions included items regarding demographic (gender, age, education level, marital status, race/ethnicity, height, weight), lifestyle (alcohol consumption, frequency and duration of aerobic and resistance training, average hours of sleep per night), and military (pay grade [rank], special operations status, military service branch) characteristics. This descriptive section was followed by a multipart question listing 31 types of caffeine-containing beverages, including coffee, teas, soft drinks, energy drinks, and caffeinated gums and medications. The latter category included Jolt Gum, Stay Alert Gum, Vivarin, No Doz Maximum, No Doz Regular, Generic Caffeine Pills, Dexatrim or other weight control aids, Bayer Headache Relief, Excedrin, and others. The survey used a standard food frequency questionnaire method in which SMs selected among names of commonly used products or could type in caffeine-containing products that were not listed. SMs were asked to provide the number of times they used the product (per day, week, or month) in the last six months and the serving size. Serving sizes were as follows: for coffee, teas and soft drinks, 8, 12, 16, 20, and ≥24 fl oz; for energy drinks, the number of cans or bottles with serving size listed for the beverage; and for gums and medications, sticks of gum or number of pills. The questionnaire also asked the SM to record the typical amount of beer, wine, and liquor they consumed. They could select either a small, medium, or large serving size for each of the three beverage types, with a medium serving size anchored at 12 oz for beer, one medium glass for wine, and one shot for liquor. They subsequently listed the number of times per day, week, month, or year they consumed the alcoholic beverage.

### 2.3. Data Analysis

Caffeine consumption (mg/day) was calculated based on the self-reported product, its serving size, and frequency of use. Sources of information on caffeine content of specific products included product and company websites and a database of caffeine in coffees, teas, sodas, and energy drinks [22]. Body mass index (BMI) was calculated as weight/height^2^ (kg/m^2^). Weekly duration of aerobic and resistance training (min/week) was calculated by multiplying weekly exercise frequency (sessions/week) by the duration of training (min/session). Alcohol consumption was estimated using standards provided by National Institutes of Health [23]. A medium drink was defined as a 12 oz beer, 5 oz glass of wine, or a shot of liquor, each containing 14 gm of alcohol. SMs were categorized into five alcohol consumption groups which included non-users and users divided into quartiles (four approximately equal groups) based on their reported daily alcohol consumption.

Statistical analysis was conducted using the Statistical Package for the Social Sciences (SPSS; Version 19.0.0, SPSS Inc., IBM Corporation, Armonk, NY, USA). Caffeine products were grouped into eight categories that included (1) coffee; (2) hot tea; (3) other tea-based beverages; (4) colas; (5) other sodas; (6) energy drinks; (7) caffeinated gums and medications; and (8) other drinks containing caffeine. All categories were combined to arrive at an aggregated caffeine intake. Definitions of these categories are shown in Table 1.

Prevalence of use ≥1 time/week (%) and standard errors (SE) were calculated. Chi-square statistics were used to examine prevalence differences across various strata of demographic (gender, age, education level, marital status, race/ethnicity, BMI), lifestyle (alcohol consumption, weekly duration of aerobic and resistance training, sleep duration) and military (rank, special operations status, military service branch) characteristics. A one-way analysis of variance (ANOVA) was used to examine differences in daily average caffeine consumption across strata of these same characteristics. Since some participants did not complete all of the questions, the number of subjects is shown for each variable. Multivariable logistic regression was used to examine associations between the dependent variable “caffeine consumer” (≥1 time/week) and independent variables that included demographic, military, and lifestyle characteristics. Six separate regression models were developed for specific caffeine sources including any caffeine, coffee, tea, cola, energy drinks, and caffeinated gums and medications. A two-way ANOVA compared overall caffeine consumption by age and gender while a one-way ANOVA with subsequent Tukey tests compared coffee and energy drink consumption by age in men and women separately. To compare differences between military services (Marine Corps vs. Navy), two-way ANOVAs (service × each demographic, lifestyle and military characteristic) were performed for each caffeine source.

## 3. Results

Of the random sample of 10,000 active-duty SMs requested from DMDC, 328 were not contacted because they were enrolled in other NHRC military survey studies [21,24]. Therefore, 9672 SMs (5810 Marines and 3862 Navy personnel) were initially contacted—9598 by postal letter and 74 without valid postal addresses who were contacted by e-mail. Of the invited SMs, 999 Marine Corps (17.2%) and 709 Navy (18.4%) personnel completed the questionnaire (17.7% total response rate). Sixteen Marine Corps and nine Navy personnel reported service in the reserves and were not considered further. This resulted in a final sample of 983 Marine Corps and 700 Navy active-duty personnel who were included in the analyses.

### 3.1. Caffeine Prevalence

Table 2 provides prevalence of reported caffeine use by demographic, lifestyle, and military characteristics. Overall, 87% of respondents reported using products containing caffeine ≥1 time per week, with coffee being the beverage most often consumed. A larger proportion of men reported drinking colas and energy drinks while a larger proportion of women reported consuming hot teas and caffeinated gums/medications. The proportion of SMs using caffeinated products, especially for coffee and colas, progressively increased with age while the prevalence of energy drink use was highest in the younger age groups (<40 years). Caffeinated gum/medication use was highest among the youngest and oldest SMs. Higher education levels were associated with higher overall frequency of using caffeinated products, especially coffee, hot tea, and cola; lower education levels were associated with higher use of other sodas and energy drinks. A larger proportion of married individuals used caffeinated products, especially coffee and cola, while a larger proportion of single individuals consumed caffeine from hot teas and other sodas. Caffeine and coffee use was highest among the White race/ethnicity and lowest among the Black race/ethnicity while Hispanic and “other” race/ethnicity were intermediate. Hot tea use was highest among the “other” race/ethnicity group while consuming colas and energy drinks was highest among Whites and lower among the “other” race/ethnicity group. Lower BMI (<25.0 kg/m^2^) was associated with a lower consumption of coffee, energy drinks, and caffeinated gums/medications, but higher consumption of hot tea.

As alcohol consumption increased so did the overall use of caffeinated products, especially coffee and colas. Energy drink use was lowest among the alcohol nondrinkers and highest among the highest alcohol consumption group with other alcohol categories showing intermediate use. Longer weekly duration of aerobic exercise was associated with a lower prevalence of cola use. Longer weekly duration of resistance training was associated with a lower overall caffeine use, especially coffee and colas, but a higher consumption of energy drinks. Shorter nightly sleep duration was associated with higher consumption of any caffeinated products, especially energy drinks, but prevalence of coffee consumption was lowest in the shortest and longest sleep duration categories. SMs reporting the shortest nightly sleep duration also had the highest use of caffeinated gums/medications.

Higher military rank was generally associated with a higher prevalence of consuming any caffeinated product; junior and senior officers reported consuming coffee more frequently than warrant officers and enlisted SMs. Prevalence of hot tea use was lowest for warrant officers and highest for senior officers, with other ranks intermediate. Prevalence of cola consumption was lowest for the junior enlisted and highest among warrant officers. Enlisted personnel and warrant officers reported consuming energy drinks more frequently than officers. Those who reported special operations assignments had a higher prevalence of consuming caffeinated products, especially energy drinks. A larger proportion of Navy personnel consumed hot teas while Marines were more likely to use energy drinks.

### 3.2. Caffeine Consumption

Table 3 provides the estimated daily consumption (mg/day) among Navy and Marine Corps caffeine consumers (≥1 time/week) by their demographic, lifestyle, and military characteristics. Caffeine content of “other drinks” were <1 mg/day and are not shown. The reported average daily caffeine consumption among consumers was 226 mg/day with coffee, energy drinks, teas (hot and others), soda (colas and others), and caffeinated gums/medications accounting for 58%, 16%, 12%, 9%, and 5% of consumption, respectively. There were 13.2% of caffeine consumers who had an overall consumption ≥400 mg/day (15.1% of men, 8.2% of women), and 17.7% of female consumers had an overall caffeine consumption of ≥300 mg/day (age range: 18–56 years). The beverages ingested by the higher caffeine consumers were similar to those of the entire group with coffee, energy drinks, teas (hot and others), soda (colas and others), and caffeinated gums/medications accounting for 58%, 16%, 13%, 6%, and 7%, respectively.

Men consumed more total caffeine than women due to a greater consumption of coffee, other teas, cola, and energy drinks; women consumed more caffeine from hot tea and caffeinated gums/medications. When total caffeine consumption was determined based on a body weight basis, consumption was similar among men and women (2.89 vs. 2.78 mg/day/kg body weight, respectively, *p* = 0.44). Caffeine consumption increased with age due to greater consumption of coffee and to a lesser extent of cola, but younger SMs consumed more caffeine from other sodas and energy drinks. Caffeine consumption from coffee increased as the education level increased, but caffeine use from other tea, soda, and energy drinks was higher among those of lower education levels. Married SMs consumed more caffeine overall, primarily due to consumption from coffee and cola, but single SMs consumed more caffeine from hot tea. Service members reporting White race/ethnicity consumed the most total caffeine, primarily from coffee and cola, while those reporting Black race/ethnicity consumed the least and Hispanic and “other” were intermediate. Black and “other” race/ethnicity consumed more caffeine from hot tea. Service members with a BMI ≥25.0 kg/m^2^ consumed more caffeine than SMs with a BMI < 25.0 kg/m^2^, primarily due to coffee and energy drink consumption. Caffeine consumed from hot tea decreased as BMI increased.

There was a positive relationship between alcohol and caffeine consumption, driven primarily by coffee and energy drink consumption. SMs who reported abstaining from alcohol consumed the least amount of caffeine while the category of SMs who reported drinking the most alcohol (gm/day) consumed the most caffeine. In addition, SMs who reported performing the shortest weekly duration of aerobic exercise consumed more caffeine from cola. Overall caffeine consumption was highest among those reporting the shortest weekly duration of resistance training due primarily to higher consumption from coffee and cola, but energy drink consumption was highest among those doing the longest weekly duration of resistance exercise. There was an inverse relationship between sleep duration and using any caffeine products. Overall caffeine consumption increased as nightly sleep duration decreased due to consumption from coffee, other teas, energy drinks, and caffeinated gums/medications.

Senior enlisted, warrant officers, and senior officers consumed more caffeine products than junior enlisted and junior officers, primarily due to differences in caffeine consumption from coffee and cola. Compared with those of other ranks, senior officers consumed more caffeine from hot tea and less from energy drinks. Special operations assignments had little relationship with caffeine consumption. Marine Corps personnel consumed more caffeine from energy drinks than Navy personnel.

### 3.3. Characteristics Independently Associated with Caffeine Use

Table 4 shows the results of the multivariable logistic regression examining characteristics associated with caffeine use ≥1 time per week. Characteristics independently associated with overall caffeine use included older age, White race/ethnicity, higher alcohol consumption, and shorter weekly duration of resistance training. Drinking coffee was independently associated with being male, older age, higher education level, race/ethnicity other than Black, higher BMI, and higher alcohol consumption. Drinking tea was independently associated with female gender and higher alcohol consumption. Drinking soda was associated with being male, older age, White and “Other” race/ethnicity, and a shorter weekly duration of resistance training. Consuming energy drinks was associated with being male, younger age, lower education level, White race/ethnicity, higher BMI, higher alcohol consumption, shorter sleep duration, and service in the Marine Corps. Caffeinated gum/medication use was independently associated with being female, higher BMI, and a longer weekly duration of aerobic training.

### 3.4. Caffeine Consumption by Age and Gender

**Figure 1:**
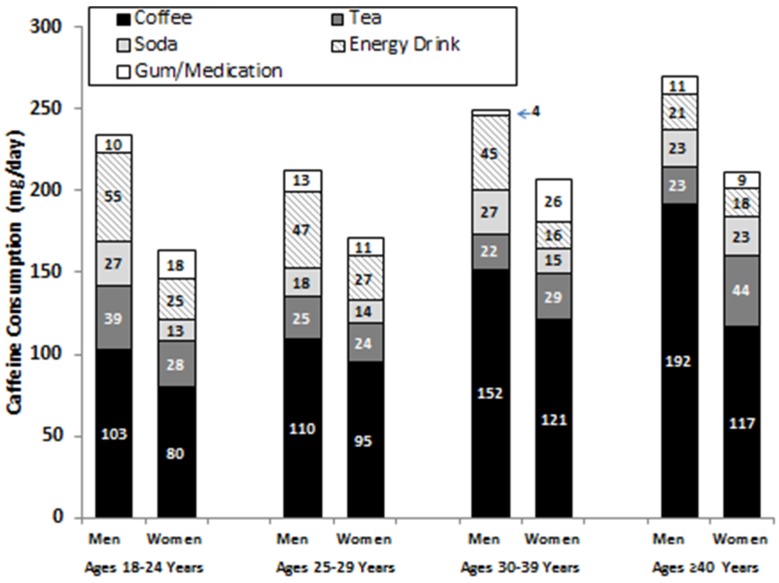
Daily average caffeine consumption of Navy and Marine Corps personnel.

Figure 1 presents daily caffeine consumption (mg/day) from all sources by age and gender. A two-way ANOVA indicated that men consumed more total caffeine than women (*p* < 0.01) and that caffeine consumption increased with age (*p* = 0.03) with no interaction between age and gender (*p* = 0.77). One-way ANOVAs with subsequent Tukey tests indicated that older men (≥40 years) consumed more caffeine from coffee (*p* < 0.01) while younger men (<40 years) ingested more caffeine from energy drinks (*p* < 0.01). Older women consumed more caffeine from coffee than younger women (*p* = 0.03), but caffeine ingestion from energy drinks was similar in younger and older women (*p* = 0.43).

### 3.5. Comparison of Navy and Marine Corps Personnel

Table 5 and Table 6 present separate analyses of the Marine Corps and Navy caffeine consumers, respectively, across the various strata of demographic, lifestyle, and military characteristics. In the 8 × 12 matrices (caffeine source × variables), there were 33 significant differences (*p* < 0.05) among Marines and 30 among Navy personnel. Of these, 21 were significant (*p* < 0.05) in both services for the same variables. Two-way ANOVAs were performed for each of the 12 variables (service × variable) for each caffeine source. Energy drinks were the only beverage that differed (*p* < 0.05) by the main effect of service, with differences found for all variables except rank (*p* = 0.59) and special operations status (*p* = 0.60).

Table 7 presents separate logistic regression models examining characteristics independently associated with caffeine use ≥1 time per week for Navy personnel. Characteristics associated with overall caffeine use included older age, ethnicity other than Black, and higher alcohol consumption. Coffee consumption was independently associated with being male, ethnicity other than Black and higher alcohol consumption. Tea consumption was independently associated with female gender while soda use was independently associated with male gender. Consuming energy drinks was associated with male gender, younger age, ethnicity other than black, single marital status, higher BMI, and higher alcohol consumption. Caffeinated gum/medication use was independently associated with female gender and higher alcohol consumption.

Table 8 presents separate logistic regression models examining characteristics associated with caffeine use ≥1 time per week for Marine Corps personnel. Characteristics associated with higher overall caffeine use included older age, White and Hispanic race/ethnicity, and higher alcohol consumption. Drinking coffee was associated with older age, higher education level, race/ethnicity exclusive of Black or “other”, higher BMI, and higher alcohol consumption. Drinking tea was independently associated only with female gender. Drinking soda was associated with male gender, older age, lower educational level, ethnicity other than Hispanic, and shorter weekly duration of resistance training. Consuming energy drinks was associated with male gender, younger age, lower education level, White race/ethnicity, higher alcohol consumption, and less sleep. Caffeinated gum/medication use was independently associated with female gender, higher BMI, longer weekly duration of aerobic training, and less sleep.

## 4. Discussion

The present study documented that 87% of Navy and Marine Corps survey respondents consume caffeine ≥1 time per week with an estimated daily consumption of 226 mg/day. Among respondents who reported regularly using caffeinated products, men consumed 32% more caffeine (mg/day) than women, and total consumption increased with age in both men and women. Coffee accounted for the most caffeine consumption overall, but younger men consumed more caffeine from energy drinks compared with older men. Consuming any caffeinated beverage was independently associated with older age, White race/ethnicity, higher alcohol consumption, and a shorter weekly duration of resistance training. Energy drink consumption was independently associated with being male, younger age, lower education level, White race/ethnicity, higher BMI, higher alcohol consumption, less sleep, and service in the Marine Corps.

### 4.1. Caffeine Prevalence and Daily Consumption

A previous study on caffeine consumption of Army personnel [20] used a questionnaire and caffeine beverage definitions that were very similar to those of the present study, although the Army study [20] used a weighted sample and different sampling technique. The present study employed a random sample while the Army study [20] enrolled soldiers at 11 installations based on location and availability. Compared with the Army investigation [20], the overall prevalence of caffeine consumption ≥1 time per week was somewhat higher in the present study (87% vs. 82%) while the daily consumption of users was lower (226 mg/day in the present study vs. 347 mg/day in the Army investigation). Compared with Navy and Marine Corps personnel, Army personnel consumed more caffeine from coffee (155 vs. 130 mg/day), cola (32 vs. 16 mg/day), other soda (30 vs. 5 mg/day), and energy drinks (97 vs. 37 mg/day). In further contrast with the Navy and Marine Corps data, the Army investigation [20] found little association between overall daily caffeine consumption and age, educational level, rank, BMI, or resistance training. The results of the two studies were similar in that men consumed more caffeine than women and those of White race/ethnicity consumed more caffeine than SMs of Black race/ethnicity.

There are four previous population-based estimates of caffeine consumption in Americans. Data from National Health and Nutrition Survey (NHANES) [1,25] was collected from a 24 h dietary recall that included foods and beverages (beverages provided 98% of caffeine consumed), but did not include medications. One study [1] using NHANES data (2001–2010) indicated that 89% of men and 89% of women consumed caffeine on any given day with an average consumption of 211 and 161 mg/day for ≥19-year-old men and women, respectively. The second study [25], using NHANES data from 2011 to 2012, found that men and women aged ≥20 years consumed an average of 196 and 151 mg/day, respectively. Caffeine intake data are also available from the US Department of Agriculture (USDA) Continuing Survey of Food Intakes by Individuals (1994 to 1996 and 1998) [19] and are based on two days of dietary intake data that included beverages and foods but did not include energy drinks and medications. These data indicated 89% of adult men and 91% of adult women (18–34 years of age) consumed caffeinated substances and averaged 199 and 166 mg/day for men and women, respectively. Another study provided corrected values for this survey based on an updated USDA nutrient database and reported caffeine consumptions of 193 and 149 mg/day for men and women ≥20 years of age, respectively [26]. Data from the Kantar Worldwide Beverage Consumption Panel (involving US consumers only) was obtained from an online, seven-day beverage consumption record and indicated that about 90% of individuals ≥18 years of age consumed caffeinated beverages with an average caffeine consumption of about 200 mg/day among caffeine users (males and females were not separated) [2]. Despite differences in methods, prevalence values were similar to the 87% prevalence observed in the present study (≥1 week) but the average consumption of 242 and 183 mg/day for men and women, respectively, was higher.

The estimated average daily caffeine consumption for Navy and Marine Corps personnel was well below the 400 mg/day for men and 300 mg/day for women of reproductive age that may be associated with adverse effects [18]. Nonetheless, the present study found about 15% of men and 18% of women exceeded these recommended amounts. Beverages from which this caffeine was consumed did not differ from that of the entire group, and it may be that these high caffeine consumers did not realize the amount of caffeine they were ingesting. Also, there are genetic differences that may influence higher caffeine consumption. A genetic polymorphism (caffeine N^3^-demethylation) allows some individuals to metabolize caffeine in the liver more rapidly than others, and another polymorphism may be associated with higher caffeine tolerance and consumption [27,28,29].

The 28% energy drink use prevalence found in the present study (≥1 time/week) was lower than the 39% reported in the Army [20], the 31% in the Air Force [30], and the 38% in a sample of SMs consisting of many professional military medical personnel and military college students [31]. Among Soldiers and Marines deployed to Afghanistan, 45% reported using energy drinks daily [32]. Studies of energy drink consumption among US college students found that 39% consumed an energy drink in the past week [33] and 36% within the past two weeks [34].

### 4.2. Characteristics Associated with Caffeine Use

In agreement with the present study, others [1,19,20,25] have reported men consumed greater amounts of caffeine than women. Nonetheless, this study found that when caffeine consumption was determined on a per kg body weight basis, men and women consumed similar amounts, as found in a study of a representative US sample [19]. Acute caffeine consumption modestly affects moods, such as vigor and fatigue, as well as hemodynamic measures (e.g., blood pressure, cardiac output) in both men and women [35,36,37], and it is plausible that both men and women consume caffeinated beverages to provide similar behavioral and/or cardiovascular effects.

Investigations involving representative civilian samples [1,2,19,25] have reported that overall caffeine consumption increases with age. In this study, coffee consumption accounted for most of the caffeine ingested in all age groups, but younger (aged <40 years) individuals consumed almost twice as much caffeine from energy drinks compared with older (aged ≥40 years) individuals (40 vs. 21 mg/day, *p* < 0.01) and were more than twice as likely to use them (30 vs. 14%, *p* < 0.01). Energy drinks are a relatively new source of caffeine introduced into the American market in 1997 [38]. Advertising of these drinks is targeted at teenagers and individuals aged 18–34 years [39] and may have influenced energy drink consumption in the younger age groups in the present study. It is likely that energy drink consumption will continue to increase in the future since the US energy drink market is projected to grow by 52% between 2014 and 2019 [40].

An interesting finding was that, compared to those of White race/ethnicity, those of Black race/ethnicity had a lower prevalence of caffeine use and a lower total consumption among consumers as reported in other investigations [20,25]. Race/ethnic differences have been reported in dietary intake [41,42]. These disparities do not appear to be explained by nutritional knowledge and briefs [42], but education level and income differences may explain some of the variance [42,43,44]. In the present study, the racial/ethnic differences in caffeine prevalence remained after controlling for education, rank (a surrogate for income), and other factors in the multivariate logistic regression. The potential reasons for the race/ethnic differences in caffeine prevalence and consumption are likely complex and may differ in the military compared to the general population.

The relationship between alcohol consumption and caffeinated beverage use has been reported elsewhere [45,46,47,48,49]. In the present investigation, both univariate and multivariable analyses showed that alcohol consumption was positively associated with caffeine use, especially the consumption of coffee and energy drinks. Studies of monozygotic and dizygotic twins suggest that there is a common genetic factor underlying this association of both alcohol and caffeine consumption, although substance-specific factors and environmental influences still seem to contribute to the variance in caffeine consumption [50,51,52]. Social and familial environmental factors appear to influence caffeine consumption in adolescence, and genetic factors become progressively more important through early and middle adulthood [52].

Epidemiological studies that have used convenience samples to examine associations between caffeine and sleep duration or sleep quality have found conflicting results [47,53,54,55,56]. On the other hand, investigations using randomized, population-based samples have shown shorter sleep duration is associated with higher caffeine consumption [32,57,58]. Since it is well known that caffeine can reduce the tendency to sleep, individuals who need to stay awake often choose to consume caffeine. In fact caffeine is sold as an FDA-approved over-the-counter drug for this purpose. The present study found in univariate analysis that sleep duration was shorter among those who used more caffeine in the form of coffee, teas, energy drinks, and caffeinated gums/medications; however, in multivariable analysis shorter sleep duration was independently associated only with use of energy drinks and gums/medication, primarily among the Marine personnel. Other investigations have demonstrated that less sleep is associated with energy drink use [32,57]. The biological basis of this association likely relates to caffeine’s primary mechanism of action—the modulation of central adenosine receptors. Adenosine is a neuromodulator that acts as a regulator of sleep and arousal [59,60]. Caffeine competes with adenosine at adenosine receptor sites to increase arousal and reduce the propensity to sleep [60,61]. Experimental studies indicate that caffeine can reduce sleep duration, likely in relation to the dosage and proximity to bedtime. Consumption of 200 mg of caffeine 16 h prior to sleep modestly shortened sleep duration (11 min) [62]; a 100 mg dose administered 3 h and 1 h prior to bedtime (200 mg total) reduced sleep duration by about 30 min [63]; and a 400 mg dose taken 6 h prior to bedtime reduced sleep duration time by 72 min [64].

A previous study of Army personnel found that aerobic exercise duration was not associated with overall caffeine consumption [20] and, in agreement, Navy and Marine Corp personnel demonstrated little relationship between caffeine consumption and aerobic exercise duration in univariate or multivariable analyses. The Army study [20] found a nonsignificant trend indicating that those participating in resistance training consumed less caffeine. In the present study, a greater duration of resistance training was independently associated with consuming less caffeine when the Navy and Marine Corps personnel were combined, but the relationship was diminished when the two groups were analyzed separately. The Army study used only two categories (yes or no) to characterize resistance training while the present study used four categories which may have more effectively quantified the training volume. In a previous study of Navy and Marine Corp personnel, dietary supplement use was strongly associated with increasing resistance training duration [65]. Many dietary supplements contain caffeine, and the caffeine content of some of these can be very high [66,67]. Accurately determining the caffeine content of dietary supplements is difficult because manufacturers are not required to list the amount of caffeine on their supplement facts labels, amounts are usually not available on company websites, and if the ingredients are proprietary the manufacturer is not required to list caffeine at all [67]. It is possible that SMs involved in resistance training consumed less caffeine from beverages to avoid adverse effects resulting from high dosages of caffeine in their dietary supplements.

In univariate analysis and in multivariable analyses in the combined group, higher BMI was associated with a higher proportion of SMs consuming coffee, energy drinks, and caffeinated gum/medications. It is possible these associations may have different explanations depending on the specific caffeine-containing product. For example, many energy drinks contain sugars and other high-calorie carbohydrates that may contribute to weight gain or weight maintenance. Some individuals may use large amounts of sugar in their coffee or tea that may have similar effects on body weight. On the other hand, caffeine has thermogenic properties that may reduce weight acutely [68] and in the longer term, but long-term effects on weight are relatively modest (<1 kg) [69]. Nonetheless, some individuals with higher BMIs may consume caffeinated beverages for their thermogenic properties. There are strict weight standards for height and body fat in the Navy and Marine Corps [70,71], and SMs who do not meet these standards can receive adverse performance reports and be discharged from service for repeated failures to achieve the standards.

### 4.3. Limitations

This study has several limitations. All data were self-reported and suffer from the usual limitations associated with this method, including non-response bias, recall bias, social desirability, errors in self-observation, and inadequate recall [72,73]. These biases could account for errors in reporting serving sizes and how many times per week caffeinated products were consumed, and thus errors in estimating the milligrams per day of caffeine consumption. Another limitation was the large number of statistical tests conducted examining relationships between caffeine use and amount and the demographic, lifestyle, and military characteristics. The more effects investigated, the greater the chance of making a Type 1 error where a null hypothesis will turn up significant. However, it is important to show these relationships and their probability levels in this investigation for more adequate comparisons with other investigations.

Caffeine data was only obtained from beverages and did not include caffeine from foods or dietary supplements. However, a previous study [1] that did include caffeine in foods and beverages found that beverages accounted for 98% of caffeine consumption, so it is likely that omitting foods had only a small effect on caffeine estimates. On the other hand, the caffeine content of some dietary supplements can be high [67], and many Navy and Marine Corps personnel use dietary supplements [65]. These factors might have led to an underestimation of the actual caffeine ingested. Finally, the caffeine content of beverages was obtained from company websites, labels, and a database of caffeinated beverages. Although company websites and food labels are likely reliable, estimates of caffeine in coffees and teas may be less so. Where the coffee beans or tea leaves are harvested and how they are processed and brewed can affect their caffeine content [74,75,76].

## 5. Conclusions

The present investigation expands knowledge on the prevalence and amount of caffeine consumed in the military services, a group largely neglected in national surveys. Among Navy and Marine Corps personnel, 87% reported using caffeinated beverages ≥1 time/week, with male and female consumers ingesting (mean ± standard error) 242 ± 7 and 183 ± 8 mg/day, respectively. The most commonly consumed caffeinated products (% using) were coffee (65%), soda (54%), tea (40%), and energy drinks (28%). The prevalence of energy drink consumption and caffeine ingested from energy drinks was about twice as high among those aged <40 years compared with those aged ≥40 years. Sources of caffeine were similar for Marine Corps and Navy personnel except that Marines obtained more caffeine from energy drinks. Characteristics independently associated with regular caffeine use included older age, white race/ethnicity, higher alcohol consumption, and shorter weekly duration of resistance training. The prevalence of caffeine use by Navy and Marine Corps personnel was similar to that reported from NHANES data, although total consumption of caffeine (mg/day) was higher in Navy and Marine Corps personnel. Characteristics associated with caffeine use in these SMs were similar to civilian investigations. Future research should focus on the caffeine use of Air Force personnel which have not been investigated to date and reasons for use of caffeine-containing products.

## Figures and Tables

**Table 1:** Caffeine categories in study of United States Navy and Marine Corps personnel.

Category	Definition
Any caffeine	All caffeine-containing beverages, gums, and medications as listed below
Coffee	Hot or cold brewed coffee, espresso, cappuccino, frozen blended coffee drinks, and other coffee-based beverages that contain caffeine
Hot tea *	Hot brewed tea of any type that contains caffeine
Other tea *	Other teas including iced tea and cold tea blends that include caffeine
Cola ^†^	All brands of cola-type beverages that contain caffeine
Other soda ^†^	Sodas that are not colas but are carbonated and contain caffeine including root beers, orange soda, and other flavored sodas
Energy drink	All beverages labeled as energy drinks of any kind that contain caffeine
Gum or medication	Chewing gums, prescription medications, weight control aids, and other over-the-counter medications that contain caffeine
Other drink	Flavored waters, hot chocolate, chocolate milks, and derivatives that contain caffeine

* Hot tea and other tea were combined for some analyses. ^†^ Cola and other soda were combined for some analyses.

**Table 2:** Prevalence (% ± SE) of reported use of caffeine (≥1 time/week) among Navy and Marine Corps personnel by demographic, lifestyle, and military characteristics. (*p*-values are from chi-square analyses).

Variable	Strata	Any Caffeine	Coffee	Hot Tea	Other Tea	Cola	Other Soda	Energy Drink	Other Drink	Gum/Medication
Group	All (*n* = 1683)	87.0 ± 0.8	65.0 ± 1.2	16.8 ± 0.9	22.8 ± 1.0	39.6 ± 1.2	14.3 ± 0.9	27.5 ± 1.1	2.9 ± 0.4	8.4 ± 0.7
Gender	Men (*n* = 1198)	87.8 ± 0.9	66.0 ± 1.4	12.7 ± 1.0	23.0 ± 1.2	43.8 ± 1.4	15.2 ± 1.0	30.9 ± 1.3	2.4 ± 0.4	6.8 ± 0.7
	Women (*n* = 485)	85.2 ± 1.6	62.5 ± 2.2	27.0 ± 2.0	22.1 ± 1.9	29.1 ± 2.1	12.0 ± 1.5	19.2 ± 1.8	3.9 ± 0.9	12.2 ± 1.5
	*p*-value	0.14	0.17	<0.01	0.64	<0.01	0.09	<0.01	0.10	<0.01
Age (years)	18–24 (*n* = 443)	76.7 ± 2.0	51.7 ± 2.4	17.4 ± 1.8	25.4 ± 2.1	30.7 ± 2.2	17.4 ± 1.8	29.6 ± 2.2	3.6 ± 0.9	9.9 ± 1.4
	25–29 (*n* = 407)	88.7 ± 1.6	63.1 ± 2.4	16.2 ± 1.8	21.1 ± 2.0	37.1 ± 2.4	12.5 ± 1.6	33.9 ± 2.3	2.2 ± 0.7	5.9 ± 1.2
	30–39 (*n* = 552)	90.6 ± 1.2	72.8 ± 1.9	15.8 ± 1.6	22.1 ± 1.8	45.5 ± 2.1	14.1 ± 1.5	27.7 ± 1.9	2.9 ± 0.7	7.6 ± 1.1
	≥40 (*n* = 280)	93.9 ± 1.4	73.6 ± 2.6	18.9 ± 2.3	22.5 ± 2.5	45.7 ± 3.0	11.8 ± 1.9	14.3 ± 2.1	2.5 ± 0.9	11.1 ± 1.9
	*p*-value	<0.01	<0.01	0.67	0.45	<0.01	0.11	<0.01	0.65	0.05
Education	Some high school/high school graduate (*n* = 393)	79.1 ± 2.1	51.9 ± 2.5	13.7 ± 1.7	23.9 ± 2.2	35.6 ± 2.4	15.5 ± 1.8	30.3 ± 2.3	3.1 ± 0.9	8.4 ± 1.4
	Some college/Associate’s degree (*n* = 729)	87.5 ± 1.2	64.6 ± 1.8	15.4 ± 1.3	23.9 ± 1.6	38.4 ± 1.8	16.3 ± 1.4	33.9 ± 1.8	2.7 ± 0.6	9.7 ± 1.1
	Bachelor’s/Graduate degree (*n* = 561)	92.0 ± 1.1	74.7 ± 1.8	20.9 ± 1.7	20.7 ± 1.7	43.9 ± 2.1	10.7 ± 1.3	17.3 ± 1.6	2.9 ± 0.7	6.6 ± 1.0
	*p*-value	<0.01	<0.01	<0.01	0.34	0.03	0.01	<0.01	0.96	0.13
Marital status	Single (*n* = 570)	82.6 ± 1.6	58.4 ± 2.1	20.4 ± 1.7	23.0 ± 1.8	31.2 ± 1.9	17.4 ± 1.6	28.1 ± 1.9	3.5 ± 0.8	8.6 ± 1.2
	Married (*n* = 1113)	89.3 ± 0.9	68.4 ± 1.4	15.0 ± 1.1	22.7 ± 1.3	43.8 ± 1.5	12.7 ± 1.0	27.2 ± 1.3	2.5 ± 0.5	8.3 ± 0.8
	*p*-value	<0.01	<0.01	0.01	0.91	<0.01	0.01	0.71	0.25	0.82
Race/ethnicity	White (*n* = 1063)	90.9 ± 0.9	69.5 ± 1.4	16.4 ± 1.1	23.4 ± 1.3	43.9 ± 1.5	15.0 ± 1.1	30.1 ± 1.4	3.0 ± 0.5	8.7 ± 0.9
	Black (*n* = 194)	77.3 ± 3.0	45.4 ± 3.6	17.0 ± 2.7	21.1 ± 2.9	30.4 ± 3.3	14.9 ± 2.6	21.6 ± 3.0	3.1 ± 1.2	10.8 ± 2.2
	Hispanic (*n* = 271)	81.9 ± 2.3	64.6 ± 2.9	12.9 ± 2.0	21.4 ± 2.5	32.5 ± 2.8	10.7 ± 1.9	24.7 ± 2.6	2.2 ± 0.9	7.4 ± 1.6
	Other (*n* = 155)	81.9 ± 3.1	59.4 ± 3.9	26.5 ± 3.5	23.2 ± 3.4	33.5 ± 3.8	14.8 ± 2.9	21.9 ± 3.3	2.6 ± 1.3	5.2 ± 1.8
	*p*-value	<0.01	<0.01	<0.01	0.84	<0.01	0.34	0.02	0.90	0.26
Body mass index (kg/m^2^)	<25.0 (*n* = 659)	85.9 ± 1.4	60.1 ± 1.9	20.5 ± 1.6	22.3 ± 1.6	36.7 ± 1.9	14.4 ± 1.4	23.1 ± 1.6	3.3 ± 0.7	7.0 ± 1.0
	25.0–29.9 (*n* = 844)	87.7 ± 1.1	69.1 ± 1.6	15.5 ± 1.2	23.0 ± 1.4	42.1 ± 1.7	14.1 ± 1.2	30.7 ± 1.6	2.4 ± 0.5	8.4 ± 1.0
	≥30.0 (*n* = 163)	89.6 ± 2.4	65.6 ± 3.7	9.2 ± 2.3	24.5 ± 3.4	40.5 ± 3.8	16.0 ± 2.9	30.7 ± 3.6	3.7 ± 1.5	13.5 ± 2.7
	*p*-value	0.37	<0.01	<0.01	0.83	0.11	0.83	<0.01	0.44	0.03
Alcohol consumption	None (*n* = 401)	75.3 ± 2.2	46.4 ± 2.5	13.0 ± 1.7	20.0 ± 2.0	32.4 ± 2.3	15.7 ± 1.8	21.2 ± 2.0	4.0 ± 1.0	8.2 ± 1.4
	<1.33 g/day (*n* = 320)	86.9 ± 1.9	61.6 ± 2.7	20.0 ± 2.2	21.6 ± 2.3	36.3 ± 2.7	17.2 ± 2.1	28.1 ± 2.5	3.8 ± 1.1	10.0 ± 1.7
	1.34–3.99 g/day (*n* = 320)	91.6 ± 1.6	70.3 ± 2.6	15.0 ± 2.0	21.6 ± 2.3	41.9 ± 2.8	11.9 ± 1.8	27.8 ± 2.5	2.2 ± 0.8	8.4 ± 1.6
	4.00–8.93 g/day (*n* = 322)	91.9 ± 1.5	75.5 ± 2.4	18.9 ± 2.2	25.2 ± 2.4	42.9 ± 2.8	11.5 ± 1.8	28.6 ± 2.5	0.9 ± 0.5	5.9 ± 1.3
	>8.93 g/day (*n* = 320)	92.5 ± 1.5	75.9 ± 2.4	18.1 ± 2.2	26.6 ± 2.5	46.3 ± 2.8	14.7 ± 2.0	33.4 ± 2.6	3.1 ± 1.0	9.4 ± 1.6
	*p*-value	<0.01	<0.01	0.07	0.20	<0.01	0.17	<0.01	0.10	0.39
Aerobic exercise duration	<101 min/week (*n* = 414)	87.2 ± 1.6	64.5 ± 2.4	16.4 ± 1.8	24.4 ± 2.1	44.2 ± 2.4	12.3 ± 1.6	28.0 ± 2.2	1.9 ± 0.7	7.5 ± 1.3
	101–180 min/week (*n* = 384)	89.3 ± 1.6	66.7 ± 2.4	14.8 ± 1.8	19.8 ± 2.0	41.7 ± 2.5	17.4 ± 1.9	25.3 ± 2.2	1.8 ± 0.7	6.5 ± 1.3
	181–290 min/week (*n* = 462)	88.3 ± 1.5	66.9 ± 2.2	18.4 ± 1.8	25.1 ± 2.0	39.2 ± 2.3	12.8 ± 1.6	28.6 ± 2.1	4.1 ± 0.9	8.2 ± 1.3
	≥291 min/week (*n* = 409)	84.8 ± 1.8	63.1 ± 2.4	17.8 ± 1.9	22.2 ± 2.1	34.0 ± 2.3	15.4 ± 1.8	28.4 ± 2.2	3.4 ± 0.9	11.5 ± 1.6
	*p*-value	0.25	0.61	0.53	0.26	0.02	0.13	0.70	0.12	0.06
Resistance training duration	<46 min/week (*n* = 402)	90.5 ± 1.5	67.9 ± 2.3	19.7 ± 2.0	23.9 ± 2.1	46.3 ± 2.5	14.9 ± 1.8	21.9 ± 2.1	2.7 ± 0.8	9.2 ± 1.4
	46–135 min/week (*n* = 470)	90.6 ± 1.3	70.6 ± 2.1	14.5 ± 1.6	25.3 ± 2.0	44.3 ± 2.3	14.7 ± 1.6	27.4 ± 2.1	3.0 ± 2.1	7.0 ± 1.2
	136–270 min/week (*n* = 395)	86.6 ± 1.7	62.5 ± 2.4	16.7 ± 1.9	21.5 ± 2.1	36.5 ± 2.4	13.9 ± 1.7	27.6 ± 2.2	3.0 ± 0.9	8.1 ± 1.4
	≥271 min/week (*n* = 389)	82.0 ± 1.9	59.6 ± 2.3	17.7 ± 1.9	21.6 ± 2.1	31.1 ± 2.3	14.4 ± 1.8	33.4 ± 2.4	2.8 ± 0.8	9.8 ± 1.5
	*p*-value	<0.01	<0.01	0.23	0.48	<0.01	0.98	0.01	0.99	0.48
Sleep duration	≤4 h/night (*n* = 65)	90.7 ± 3.6	52.3 ± 6.2	12.3 ± 4.1	26.2 ± 5.5	38.5 ± 6.0	16.9 ± 4.6	49.2 ± 6.2	3.1 ± 2.1	16.9 ± 4.6
	5–6 h/night (*n* = 817)	89.4 ± 1.1	67.8 ± 1.6	15.9 ± 1.3	21.6 ± 1.4	42.5 ± 1.7	14.1 ± 1.2	32.2 ± 1.6	2.7 ± 0.6	9.8 ± 1.0
	7–8 h/night (*n* = 716)	86.6 ± 1.3	64.5 ± 1.8	18.7 ± 1.5	23.7 ± 1.6	38.1 ± 1.8	14.1 ± 1.3	21.4 ± 1.5	2.8 ± 0.6	6.0 ± 0.9
	≥9 h/night (*n* = 28)	75.0 ± 8.2	53.6 ± 9.4	10.7 ± 5.8	25.0 ± 8.2	25.0 ± 8.2	17.9 ± 7.2	21.4 ± 7.8	7.1 ± 4.9	10.7 ± 5.8
	*p*-value	0.05	0.03	0.26	0.72	0.12	0.87	<0.01	0.58	<0.01
Rank	Junior enlisted (*n* = 442)	76.2 ± 2.0	50.0 ± 2.4	17.0 ± 1.8	25.9 ± 2.1	29.0 ± 2.2	17.2 ± 1.8	29.4 ± 2.2	3.8 ± 0.9	8.6 ± 1.3
	Senior enlisted (*n* = 786)	89.7 ± 1.1	67.2 ± 1.7	15.1 ± 1.3	22.3 ± 1.5	41.1 ± 1.8	14.5 ± 1.3	32.6 ± 1.7	1.9 ± 0.5	9.7 ± 1.1
	Warrant officer (*n* = 38)	89.5 ± 5.0	65.8 ± 7.7	7.9 ± 4.4	18.4 ± 6.3	50.0 ± 8.1	13.2 ± 5.5	28.9 ± 7.4	7.9 ± 4.4	7.9 ± 4.4
	Junior officer (*n* = 235)	91.1 ± 1.9	77.4 ± 2.7	18.3 ± 2.5	19.1 ± 2.6	45.5 ± 3.2	11.1 ± 2.0	18.7 ± 2.5	2.1 ± 0.9	4.7 ± 1.4
	Senior officer (*n* = 182)	96.2 ± 1.4	75.8 ± 3.2	23.9 ± 3.2	24.2 ± 3.2	48.9 ± 3.7	10.4 ± 2.3	12.1 ± 2.4	4.4 ± 1.5	7.1 ± 1.9
	*p*-value	<0.01	<0.01	0.04	0.35	<0.01	0.12	<0.01	0.06	0.18
Special Operations	No (*n* = 1632)	86.9 ± 0.8	65.1 ± 1.2	16.9 ± 0.9	22.6 ± 1.0	39.5 ± 1.2	14.3 ± 0.9	27.3 ± 1.1	2.8 ± 0.4	8.5 ± 0.7
	Yes (*n* = 41)	97.6 ± 2.4	70.7 ± 7.1	12.2 ± 5.1	31.7 ± 7.3	46.3 ± 7.8	14.6 ± 5.5	41.5 ± 7.7	4.9 ± 3.4	2.4 ± 2.4
	*p*-value	0.04	0.46	0.43	0.17	0.38	0.95	0.05	0.44	0.17
Service	Navy (*n* = 700)	88.4 ± 1.2	66.9 ± 1.8	20.6 ± 1.5	24.6 ± 1.6	41.6 ± 1.9	13.7 ± 1.3	21.1 ± 1.5	2.7 ± 0.6	9.1 ± 1.1
	Marine Corps (*n* = 983)	86.1 ± 1.1	63.7 ± 1.5	14.1 ± 1.1	21.6 ± 1.3	38.1 ± 1.5	14.6 ± 1.1	32.0 ± 1.5	3.0 ± 0.5	7.8 ± 0.9
	*p*-value	0.15	0.18	<0.01	0.15	0.16	0.59	<0.01	0.77	0.34

Abbreviation: SE = standard error.

**Table 3:** Caffeine consumption (mean ± SE mg/day) among Navy and Marine Corps consumers (≥1 time/week) by demographic, lifestyle, and military characteristics. (*p*-values are from one-way analysis of variance).

Variable	Strata	Any Caffeine	Coffee	Hot Tea	Other Tea	Cola	Other Soda	Energy Drink	Gum/Medication
Group	All (*n* = 1465)	226 ± 5	130 ± 4	8 ± 1	18 ± 2	16 ± 1	5 ± 1	37 ± 2	11 ± 2
Gender	Men (*n* = 1052)	242 ± 7	142 ± 5	5 ± 1	21 ± 2	18 ± 1	6 ± 1	43 ± 2	9 ± 2
	Women (*n* = 413)	183 ± 8	99 ± 6	17 ± 2	12 ± 2	11 ± 1	4 ± 1	23 ± 3	17 ± 3
	*p*-value	<0.01	<0.01	<0.01	0.03	<0.01	0.28	<0.01	0.04
Age (years)	18–24 (*n* = 340)	203 ± 12	93 ± 8	10 ± 2	24 ± 5	12 ± 1	9 ± 2	42 ± 4	14 ± 3
	25–29 (*n* = 361)	201 ± 11	106 ± 7	8 ± 1	16 ± 4	13 ± 1	4 ± 1	41 ± 4	12 ± 6
	30–39 (*n* = 500)	241 ± 8	146 ± 7	7 ± 1	17 ± 3	19 ± 2	5 ± 1	39 ± 3	9 ± 2
	≥40 (*n* = 263)	261 ± 13	180 ± 11	10 ± 2	16 ± 3	19 ± 2	4 ± 1	21 ± 4	10 ± 3
	*p*-value	<0.01	<0.01	0.16	0.40	<0.01	0.03	<0.01	0.79
Education	Some high school/high school graduate (*n* = 311)	213 ± 12	100 ± 9	9 ± 2	24 ± 5	14 ± 2	9 ± 2	46 ± 5	12 ± 3
	Some college/Associate’s degree (*n* = 638)	226 ± 8	122 ± 6	8 ± 1	21 ± 3	15 ± 1	6 ± 1	43 ± 3	12 ± 2
	Bachelor’s/Graduate degree (*n* = 516)	233 ± 9	157 ± 7	9 ± 1	11 ± 2	19 ± 2	2 ± 1	24 ± 3	10 ± 2
	*p*-value	0.39	<0.01	0.47	0.02	0.08	<0.01	<0.01	0.95
Marital status	Single (*n* = 471)	209 ± 9	107 ± 7	12 ± 2	20 ± 4	13 ± 1	7 ± 1	37 ± 3	13 ± 3
	Married (*n* = 994)	234 ± 6	140 ± 5	7 ± 1	17 ± 2	17 ± 1	5 ± 1	37 ± 3	10 ± 5
	*p*-value	0.03	<0.01	<0.01	0.41	0.03	0.16	0.99	0.57
Race/ethnicity	White (*n* = 966)	249 ± 7	147 ± 5	8 ± 1	18 ± 2	19 ± 1	6 ± 1	40 ± 3	12 ± 3
	Black (*n* = 150)	150 ± 14	69 ± 8	11 ± 2	15 ± 5	10 ± 2	5 ± 2	29 ± 5	10 ± 3
	Hispanic (*n* = 222)	196 ± 12	106 ± 7	7 ± 1	21 ± 6	11 ± 2	2 ± 1	36 ± 5	11 ± 3
	Other (*n* = 127)	189 ± 14	109 ± 12	14 ± 3	15 ± 3	11 ± 3	6 ± 2	27 ± 5	7 ± 5
	*p*-value	<0.01	<0.01	0.04	0.78	<0.01	0.31	0.14	0.90
Body mass index (kg/m^2^)	<25.0 (*n* = 566)	198 ± 8	112 ± 6	11 ± 1	17 ± 3	15 ± 1	7 ± 1	28 ± 3	7 ± 2
	25.0–29.9 (*n* = 740)	247 ± 8	143 ± 6	7 ± 1	19 ± 3	16 ± 1	4 ± 1	43 ± 3	13 ± 3
	≥30.0 (*n* = 146)	230 ± 17	132 ± 13	4 ± 1	15 ± 3	19 ± 3	4 ± 1	43 ± 7	14 ± 4
	*p*-value	<0.01	<0.01	<0.01	0.71	0.56	0.14	<0.01	0.29
Alcohol consumption	None (*n* = 302)	192 ± 12	93 ± 8	7 ± 1	16 ± 3	18 ± 2	7 ± 2	31 ± 4	19 ± 8
	<1.34 g/day (*n* = 278)	208 ± 11	107 ± 8	13 ± 2	18 ± 4	15 ± 2	4 ± 1	35 ± 5	13 ± 3
	1.34–3.99 g/day (*n* = 293)	229 ± 13	141 ± 11	5 ± 1	21 ± 5	14 ± 2	4 ± 2	36 ± 4	10 ± 3
	4.00–8.93 g/day (*n* = 296)	237 ± 11	147 ± 8	8 ± 1	15 ± 3	15 ± 2	4 ± 1	38 ± 4	9 ± 3
	>8.93 g/day (*n* = 296)	263 ± 12	159 ± 9	9 ± 2	21 ± 4	18 ± 2	5 ± 1	47 ± 5	5 ± 2
	*p*-value	<0.01	<0.01	<0.01	0.76	0.43	0.22	0.02	0.20
Aerobic exercise duration	<101 min/week (*n* = 361)	225 ± 10	129 ± 8	8 ± 1	16 ± 3	19 ± 2	5 ± 2	40 ± 5	7 ± 2
	101–180 min/week (*n* = 343)	225 ± 10	131 ± 8	8 ± 2	18 ± 4	19 ± 2	6 ± 1	34 ± 4	9 ± 2
	181–290 min/week (*n* = 408)	234 ± 10	139 ± 8	9 ± 1	21 ± 4	14 ± 1	5 ± 1	37 ± 4	10 ± 2
	≥291 min/week (*n* = 347)	219 ± 12	119 ± 8	8 ± 1	17 ± 3	12 ± 1	5 ± 1	37 ± 4	19 ± 7
	*p*-value	0.79	0.39	0.95	0.71	<0.01	0.88	0.79	0.11
Resistance training duration	<46 min/week (*n* = 364)	255 ± 11	157 ± 10	10 ± 2	17 ± 3	21 ± 2	7 ± 2	33 ± 4	10 ± 2
	46–135 min/week (*n* = 426)	215 ± 9	126 ± 7	7 ± 1	19 ± 4	16 ± 1	5 ± 1	35 ± 4	7 ± 2
	136–270 min/week (*n* = 342)	213 ± 10	128 ± 9	9 ± 2	15 ± 3	15 ± 2	4 ± 1	34 ± 4	9 ± 3
	≥271 min/week (*n* = 319)	222 ± 12	106 ± 7	8 ± 1	22 ± 5	11 ± 1	6 ± 1	48 ± 5	21 ± 7
	*p*-value	0.02	<0.01	0.60	0.60	<0.01	0.45	0.03	0.06
Sleep duration	≤4 h/night (*n* = 55)	372 ± 46	149 ± 35	13 ± 6	67 ± 24	25 ± 6	10 ± 7	72 ± 13	35 ± 13
	5–6 h/night (*n* = 730)	243 ± 8	138 ± 6	8 ± 1	19 ± 3	16 ± 1	6 ± 1	43 ± 3	13 ± 2
	7–8 h/night (*n* = 620)	191 ± 6	118 ± 5	8 ± 1	13 ± 2	16 ± 1	4 ± 1	28 ± 3	4 ± 1
	≥9 h/night (*n* = 21)	148 ± 25	75 ± 20	11 ± 9	7 ± 2	9 ± 4	17 ± 8	28 ± 10	1 ± 1
	*p*-value	<0.01	0.03	0.52	<0.01	0.18	0.05	<0.01	<0.01
Rank	Junior enlisted (*n* = 337)	197 ± 11	93 ± 8	10 ± 2	23 ± 5	11 ± 1	8 ± 2	39 ± 4	12 ± 3
	Senior enlisted (*n* = 705)	235 ± 8	126 ± 6	8 ± 1	20 ± 3	16 ± 1	6 ± 1	44 ± 3	15 ± 4
	Warrant officer (*n* = 34)	263 ± 39	149 ± 26	5 ± 3	16 ± 9	24 ± 7	5 ± 3	40 ± 14	25 ± 17
	Junior officer (*n* = 214)	210 ± 11	145 ± 9	5 ± 1	7 ± 1	17 ± 2	2 ± 1	31 ± 6	3 ± 1
	Senior officer (*n* = 175)	256 ± 13	191 ± 12	14 ± 3	14 ± 3	22 ± 3	3 ± 2	10 ± 3	3 ± 1
	*p*-value	<0.01	<0.01	<0.01	0.06	<0.01	0.05	<0.01	0.09
Special Operations	No (*n* = 1419)	227 ± 5	130 ± 4	8 ± 1	18 ± 2	16 ± 1	5 ± 1	37 ± 2	11 ± 2
	Yes (*n* = 40)	198 ± 23	120 ± 22	5 ± 2	10 ± 3	17 ± 5	1 ± 0	41 ± 10	4 ± 4
	*p*-value	0.38	0.69	0.49	0.45	0.82	0.22	0.72	0.52
Service	Navy (*n* = 619)	217 ± 7	136 ± 6	10 ± 1	15 ± 2	17 ± 1	4 ± 1	29 ± 3	7 ± 1
	Marine Corps (*n* = 846)	232 ± 7	125 ± 5	7 ± 1	21 ± 3	15 ± 1	6 ± 1	43 ± 2	14 ± 3
	*p*-value	0.14	0.17	0.09	0.09	0.42	0.06	<0.01	0.07

Abbreviations: SE = standard error.

**Table 4:** Characteristics associated with use (≥1 time/week) of specific caffeine products among Navy and Marine Corps personnel. Multivariable logistic regression was used. Data are presented as odds ratios adjusted for other factors listed with 95% confidence intervals. The odds ratio 1.00 represents the reference group for each variable.

Variable	Strata	Caffeine Beverage or Gum/Medication Consumed ≥1 Time/Week					
		Any Caffeine	Coffee	Tea *	Soda ^†^	Energy Drink	Gum/Medication
		(Model 1)	(Model 2)	(Model 3)	(Model 4)	(Model 5)	(Model 6)
Gender	Men	1.00	1.00	1.00	1.00	1.00	1.00
	Women	0.83 (0.57–1.20)	0.72 (0.55–0.94)	1.47 (1.14–1.91)	0.60 (0.46–0.77)	0.57 (0.42–0.78)	2.60 (1.67–4.06)
Age (years)	18–24	1.00	1.00	1.00	1.00	1.00	1.00
	25–29	2.01 (1.27–3.18)	1.19 (0.86–1.65)	0.96 (0.69–1.32)	1.09 (0.79–1.49)	1.28 (0.91–1.80)	0.63 (0.35–1.13)
	30–39	2.28 (1.40–3.70)	1.76 (1.25–2.49)	0.97 (0.70–1.35)	1.47 (1.06–2.02)	0.92 (0.64–1.32)	1.00 (0.57–1.74)
	≥40	3.30 (1.67–6.55)	1.56 (1.02–2.40)	1.06 (0.71–1.60)	1.23 (0.83–1.82)	0.43 (0.27–0.71)	1.70 (0.88–3.28)
Education	Some high school/high school graduate	1.00	1.00	1.00	1.00	1.00	1.00
	Some college	1.09 (0.74–1.61)	1.29 (0.96–1.72)	0.98 (0.73–1.31)	0.87 (0.66–1.16)	1.27 (0.93–1.73)	1.21 (0.74–2.00)
	College degree	1.18 (0.70–2.00)	1.73 (1.21–2.48)	0.86 (0.61–1.21)	0.81 (0.58–1.13)	0.62 (0.42–0.91)	0.77 (0.41–1.43)
Marital status	Single	1.00	1.00	1.00	1.00	1.00	1.00
	Married	1.20 (0.84–1.71)	1.17 (0.91–1.52)	0.99 (0.77–1.27)	1.18 (0.93–1.50)	0.90 (0.69–1.18)	1.10 (0.72–1.68)
Race/ethnicity	White	1.00	1.00	1.00	1.00	1.00	1.00
	Black	0.31 (0.20–0.49)	0.34 (0.24–0.48)	0.96 (0.68–1.36)	0.70 (0.50–0.98)	0.52 (0.35–0.79)	0.86 (0.50–1.48)
	Hispanic	0.56 (0.37–0.86)	1.00 (0.72–1.36)	0.89 (0.66–1.21)	0.61 (0.46–0.83)	0.56 (0.40–0.78)	0.74 (0.43–1.28)
	Other	0.47 (0.28–0.78)	0.68 (0.47–1.00)	1.39 (0.97–2.00)	0.76 (0.53–1.09)	0.62 (0.40–0.96)	0.52 (0.24–1.11)
Body mass index (kg/m^2^)	<25.0	1.00	1.00	1.00	1.00	1.00	1.00
	25.0–29.9	0.94 (0.66–1.35)	1.49 (1.16–1.91)	0.94 (0.74–1.19)	0.93 (0.73–1.17)	1.40 (1.08–1.83)	1.43 (0.93–2.21)
	≥30.0	1.37 (0.70–2.68)	1.40 (0.91–2.14)	0.84 (0.56–1.26)	0.81 (0.55–1.19)	1.55 (1.00–2.41)	2.22 (1.19–4.16)
Alcohol consumption	None	1.00	1.00	1.00	1.00	1.00	1.00
	<1.33 g/day	1.73 (1.12–2.70)	1.53 (1.11–2.13)	1.22 (0.88–1.70)	1.07 (0.78–1.47)	1.75 (1.20–2.55)	1.29 (0.74–2.25)
	1.33–3.99 g/day	2.52 (1.53–4.13)	2.20 (1.57–3.09)	1.15 (0.82–1.61)	1.10 (0.80–1.52)	1.40 (0.96–2.04)	1.18 (0.66–2.09)
	4.00–8.91 g/day	2.46 (1.48–4.08)	2.65 (1.86–3.76)	1.52 (1.09–2.13)	0.98 (0.71–1.35)	1.60 (1.09–2.35)	0.88 (0.47–1.65)
	≥8.92 g/day	3.26 (1.90–5.59)	3.07 (2.14–4.39)	1.33 (0.94–1.87)	1.30 (0.94–1.80)	1.82 (1.25–2.66)	1.57 (0.89–2.76)
Aerobic exercise duration	<101 min/week	0.82 (0.54–1.33)	0.82 (0.58–1.16)	1.00 (0.72–1.38)	1.17 (0.86–1.61)	1.03 (0.72–1.47)	0.57 (0.33–0.99)
	101–180 min/week	0.95 (0.58–1.56)	0.80 (0.57–1.13)	0.84 (0.60–1.17)	1.21 (0.88–1.66)	0.94 (0.65–1.35)	0.53 (0.30–0.94)
	181–290 min/week	0.92 (0.57–1.47)	0.89 (0.64–1.24)	1.11 (0.81–1.52)	1.05 (0.77–1.42)	0.99 (0.70–1.39)	0.72 (0.43–1.19)
	≥291 min/week	1.00	1.00	1.00	1.00	1.00	1.00
Resistance training duration	<46 min/week	1.69 (1.04–2.76)	1.07 (0.74–1.53)	0.99 (0.70–1.39)	1.60 (1.14–2.23)	0.78 (0.54–1.14)	0.98 (0.55–1.75)
	46–135 min/week	1.70 (1.05–2.77)	1.39 (0.99–1.96)	0.92 (0.66–1.27)	1.55 (1.13–2.12)	1.08 (0.76–1.53)	0.75 (0.43–1.33)
	136–270 min/week	1.22 (0.77–1.93)	0.99 (0.70–1.39)	0.82 (0.59–1.15)	1.14 (0.83–1.58)	0.95 (0.67–1.36)	0.88 (0.50–1.55)
	≥271 min/week	1.00	1.00	1.00	1.00	1.00	1.00
Sleep duration	≤4 h/night	1.51 (0.46–4.90)	0.82 (0.31–2.16)	1.48 (0.55–4.00)	2.59 (0.96–7.00)	3.18 (1.08–9.38)	1.70 (0.41–7.04)
	5–6 h/night	1.90 (0.72–5.01)	1.30 (0.57–2.98)	1.27 (0.54–2.97)	2.14 (0.91–5.06)	1.65 (0.63–4.30)	0.98 (0.28–3.46)
	7–8 h/night	1.00	1.00	1.00	1.00	1.00	1.00
	≥9 h/night	1.30 (0.49–3.43)	1.06 (0.46–2.43)	1.38 (0.59–3.24)	1.70 (0.72–4.02)	0.92 (0.35–2.41)	0.56 (0.15–2.00)
Service	Marine Corps	1.02 (0.72–1.46)	0.99 (0.78–1.27)	0.80 (0.64–1.01)	0.94 (0.75–1.17)	1.71 (1.32–2.22)	0.83 (0.56–1.24)
	Navy	1.00	1.00	1.00	1.00	1.00	1.00
Nagelkerke *R*^2^		0.15	0.16	0.03	0.08	0.16	0.09

* Includes hot and other teas. ^†^ Includes cola-type beverages and other sodas.

**Table 5:** Caffeine consumption (mean ± SE mg/day) of Marine Corps consumers (≥1 time/week) by demographic, lifestyle, and military characteristics (*p*-values are from one-way analysis of variance).

Variable	Strata	Any Caffeine	Coffee	Hot Tea	Other Tea	Cola	Other Soda	Energy Drink	Gum/Medication
Group	All (*n* = 846)	232 ± 7	125 ± 5	7 ± 1	21 ± 3	15 ± 1	6 ± 1	43 ± 3	14 ± 3
Gender	Men (*n* = 605)	251 ± 9	137 ± 6	5 ± 1	24 ± 4	18 ± 1	7 ± 1	49 ± 4	11 ± 4
	Women (*n* = 241)	188 ± 12	93 ± 8	15 ± 2	14 ± 4	9 ± 1	6 ± 2	28 ± 4	22 ± 5
	*p-*value	<0.01	<0.01	<0.01	0.10	<0.01	0.75	<0.01	0.10
Age (years)	18–24 (*n* = 237)	214 ± 14	94 ± 9	10 ± 2	26 ± 6	12 ± 2	10 ± 3	45 ± 5	16 ± 4
	25–29 (*n* = 212)	212 ± 16	109 ± 10	6 ± 2	19 ± 6	13 ± 2	3 ± 1	44 ± 6	17 ± 10
	30–39 (*n* = 285)	248 ± 12	140 ± 9	6 ± 1	18 ± 4	19 ± 2	7 ± 2	49 ± 5	11 ± 3
	≥40 (*n* = 112)	270 ± 20	181 ± 17	9 ± 3	21 ± 6	18 ± 3	4 ± 2	23 ± 7	13 ± 5
	*p-*value	0.04	<0.01	0.19	0.64	0.08	0.07	0.04	0.89
Education	Some high school/high school graduate (*n* = 230)	221 ± 15	101 ± 10	8 ± 2	29 ± 6	14 ± 2	10 ± 3	46 ± 5	13 ± 4
	Some college/Associate’s degree (*n* = 382)	230 ± 11	119 ± 8	7 ± 1	21 ± 4	15 ± 2	7 ± 2	48 ± 4	13 ± 3
	Bachelor’s/Graduate degree (*n* = 234)	247 ± 15	158 ± 10	8 ± 2	12 ± 3	18 ± 2	2 ± 1	32 ± 6	17 ± 10
	*p-*value	0.44	<0.01	0.93	0.07	0.43	0.02	0.07	0.89
Marital status	Single (*n* = 256)	216 ± 13	104 ± 9	12 ± 2	25 ± 6	12 ± 2	7 ± 2	41 ± 5	15 ± 4
	Married (*n* = 590)	240 ± 9	134 ± 6	5 ± 1	19 ± 3	17 ± 1	6 ± 1	45 ± 4	14 ± 4
	*p-*value	0.14	<0.01	<0.01	0.27	0.02	0.53	0.53	0.87
Race/ethnicity	White (*n* = 533)	257 ± 10	143 ± 7	6 ± 1	20 ± 3	18 ± 36	7 ± 1	48 ± 4	14 ± 5
	Black (*n* = 80)	178 ± 23	84 ± 13	8 ± 3	20 ± 9	10 ± 3	6 ± 5	35 ± 8	15 ± 6
	Hispanic (*n* = 166)	200 ± 14	104 ± 9	9 ± 2	24 ± 8	12 ± 2	2 ± 1	34 ± 5	14 ± 4
	Other (*n* = 67)	182 ± 17	80 ± 12	11 ± 3	21 ± 5	11 ± 3	10 ± 4	38 ± 8	12 ± 9
	*p-*value	<0.01	<0.01	0.43	0.94	0.02	0.25	0.18	0.99
Body mass index (kg/m^2^)	<25.0 (*n* = 355)	202 ± 11	105 ± 8	9 ± 1	23 ± 4	15 ± 2	9 ± 2	33 ± 4	10 ± 3
	25.0–29.9 (*n* = 440)	259 ± 11	142 ± 7	6 ± 1	20 ± 4	16 ± 1	5 ± 1	51 ± 4	18 ± 6
	≥30.0 (*n* = 42)	217 ± 32	109 ± 22	3 ± 1	18 ± 8	16 ± 5	1 ± 1	55 ± 16	15 ± 7
	*p-*value	<0.01	<0.01	0.21	0.88	0.95	0.10	<0.01	0.47
Alcohol consumption	None (*n* = 195)	190 ± 17	86 ± 10	4 ± 1	19 ± 5	17 ± 3	9 ± 2	30 ± 5	26 ± 12
	<1.34 g/day(*n* = 161)	213 ± 15	103 ± 10	13 ± 3	20 ± 7	10 ± 2	8 ± 3	42 ± 7	16 ± 5
	1.34–3.99 g/day (*n* = 167)	237 ± 18	138 ± 14	5 ± 1	24 ± 7	17 ± 3	5 ± 1	40 ± 6	10 ± 3
	4.00–8.93 g/day (*n* = 155)	258 ± 17	145 ± 12	8 ± 2	21 ± 6	16 ± 3	6 ± 3	49 ± 6	14 ± 5
	>8.93 g/day (*n* = 168)	270 ± 15	159 ± 12	9 ± 2	21 ± 5	17 ± 3	4 ± 1	58 ± 8	2 ± 1
	*p-*value	<0.01	<0.01	<0.01	0.99	0.21	0.51	0.02	0.14
Aerobic exercise duration	<101 min/week (*n* = 191)	234 ± 15	126 ± 10	7 ± 2	17 ± 5	18 ± 2	8 ± 3	50 ± 7	6 ± 3
	101–180 min/week (*n* = 192)	237 ± 14	130 ± 11	5 ± 1	24 ± 8	16 ± 3	7 ± 2	42 ± 6	13 ± 4
	181–290 min/week (*n* = 230)	238 ± 14	129 ± 11	9 ± 2	22 ± 5	16 ± 2	5 ± 2	44 ± 5	13 ± 4
	≥291 min/week (*n* = 229)	224 ± 16	117 ± 10	8 ± 2	20 ± 5	12 ± 2	5 ± 2	39 ± 5	23 ± 10
	*p-*value	0.89	0.80	0.32	0.85	0.16	0.65	0.58	0.28
Resistance training duration	<46 min/week (*n* = 173)	273 ± 19	155 ± 15	9 ± 2	23 ± 6	21 ± 3	11 ± 3	42 ± 7	12 ± 4
	46–135 min/week (*n* = 222)	225 ± 14	123 ± 10	5 ± 1	25 ± 7	14 ± 2	6 ± 2	43 ± 6	9 ± 3
	136–270 min/week (*n* = 219)	208 ± 12	122 ± 10	8 ± 2	12 ± 2	15 ± 2	4 ± 1	35 ± 4	11 ± 3
	≥271 min/week (*n* = 224)	235 ± 16	107 ± 8	9 ± 2	24 ± 6	12 ± 2	6 ± 2	52 ± 6	25 ± 11
	*p-*value	0.03	0.02	0.22	0.26	0.03	0.21	0.22	0.21
Sleep duration	≤4 h/night (*n* = 41)	378 ± 53	151 ± 41	15 ± 8	67 ± 26	23 ± 6	12 ± 9	71 ± 15	39 ± 17
	5–6 h/night (*n* = 437)	249 ± 10	132 ± 7	7 ± 1	22 ± 4	15 ± 1	7 ± 2	52 ± 4	15 ± 3
	7–8 h/night (*n* = 333)	187 ± 9	111 ± 7	8 ± 1	14 ± 3	16 ± 2	4 ± 1	30 ± 4	4 ± 2
	≥9 h/night (*n* = 10)	141 ± 44	63 ± 19	0 ± 0	7 ± 3	7 ± 5	27 ± 15	35 ± 10	3 ± 3
	*p-*value	<0.01	0.08	0.14	<0.01	0.36	0.05	<0.01	<0.01
Rank	Junior enlisted (*n* = 222)	206 ± 15	89 ± 10	10 ± 2	30 ± 7	11 ± 2	9 ± 2	45 ± 5	13 ± 4
	Senior enlisted (*n* = 424)	243 ± 11	124 ± 7	7 ± 1	21 ± 4	17 ± 2	7 ± 2	48 ± 4	19 ± 6
	Warrant officer (*n* = 30)	283 ± 43	163 ± 28	6 ± 4	15 ± 10	24 ± 8	5 ± 4	44 ± 16	27 ± 19
	Junior officer (*n* = 104)	217 ± 17	154 ± 14	5 ± 1	6 ± 1	13 ± 2	2 ± 1	37 ± 10	1 ± 1
	Senior officer (*n* = 66)	253 ± 20	187 ± 19	11 ± 5	14 ± 8	23 ± 5	1 ± 1	18 ± 60	1 ± 1
	*p-*value	0.13	<0.01	0.30	0.12	0.02	0.25	0.08	0.23
Special Operations	No (*n* = 827)	233 ± 8	125 ± 5	7 ± 1	21 ± 3	15 ± 1	6 ± 1	44 ± 3	14 ± 3
	Yes (*n* = 16)	216 ± 48	154 ± 42	7 ± 5	9 ± 5	21 ± 7	1 ± 1	24 ± 10	0 ± 0
	*p-*value	0.75	0.45	0.99	0.54	0.50	0.43	0.36	0.53

Abbreviation: SE=standard error

**Table 6:** Caffeine consumption (mean ± SE mg/day) of Navy consumers (≥1 time/week) by demographic, lifestyle, and military characteristics (*p*-values are from one-way analysis of variance).

Variable	Strata	Any Caffeine	Coffee	Hot Tea	Other Tea	Cola	Other Soda	Energy Drink	Gum/Medication
Group	All (*n* = 619)	217 ± 7	136 ± 6	10 ± 1	15 ± 1	17 ± 1	4 ± 1	29 ± 3	7 ± 1
Gender	Men (*n* = 447)	232 ± 9	148 ± 8	6 ± 1	16 ± 3	19 ± 2	5 ± 1	33 ± 3	6 ± 1
	Women (*n* = 172)	176 ± 10	106 ± 8	20 ± 3	9 ± 2	12 ± 2	2 ± 1	16 ± 3	11 ± 3
	*p–*value	<0.01	<0.01	<0.01	0.15	0.04	0.07	<0.01	0.10
Age (years)	18–24 (*n* = 103)	179 ± 20	90 ± 13	11 ± 3	18 ± 8	11 ± 2	6 ± 2	34 ± 7	9 ± 3
	25–29 (*n* = 149)	184 ± 12	101 ± 9	10 ± 2	13 ± 3	13 ± 2	5 ± 2	37 ± 6	6 ± 3
	30–39 (*n* = 215)	231 ± 12	153 ± 11	8 ± 2	15 ± 4	21 ± 3	2 ± 1	25 ± 4	6 ± 2
	≥40 (*n* = 151)	217 ± 7	179 ± 15	11 ± 3	12 ± 2	20 ± 3	4 ± 2	19 ± 5	9 ± 3
	*p–*value	<0.01	<0.01	0.72	0.80	0.03	0.20	0.07	0.81
Education	Some high school/high school graduate (*n* = 81)	188 ± 22	96 ± 17	12 ± 4	9 ± 2	15 ± 5	5 ± 2	43 ± 10	7 ± 3
	Some college/Associate’s degree (*n* = 256)	220 ± 12	127 ± 10	8 ± 2	21 ± 5	15 ± 2	5 ± 1	36 ± 4	9 ± 2
	Bachelor’s/Graduate degree (*n* = 282)	222 ± 10	156 ± 9	11 ± 2	11 ± 1	19 ± 2	2 ± 1	17 ± 3	5 ± 2
	*p–*value	0.32	<0.01	0.41	0.06	0.25	0.16	<0.01	0.38
Marital status	Single (*n* = 215)	201 ± 13	111 ± 10	12 ± 2	14 ± 4	15 ± 2	6 ± 2	33 ± 4	10 ± 3
	Married (*n* = 404)	225 ± 9	150 ± 8	9 ± 1	15 ± 2	18 ± 2	3 ± 1	26 ± 3	6 ± 1
	*p–*value	0.12	<0.01	0.15	0.92	0.35	0.04	0.20	0.10
Race/ethnicity	White (*n* = 433)	240 ± 9	152 ± 8	9 ± 1	16 ± 3	19 ± 2	4 ± 1	30 ± 3	9 ± 2
	Black (*n* = 70)	117 ± 12	52 ± 8	14 ± 4	10 ± 3	11 ± 3	5 ± 1	22 ± 6	4 ± 2
	Hispanic (*n* = 56)	184 ± 17	112 ± 14	3 ± 1	13 ± 3	10 ± 2	2 ± 1	40 ± 9	3 ± 2
	Other (*n* = 60)	197 ± 24	142 ± 21	18 ± 6	7 ± 2	12 ± 5	1 ± 1	15 ± 6	2 ± 2
	*p–*value	<0.01	<0.01	0.02	0.55	0.07	0.57	0.13	0.23
Body mass index (kg/m^2^)	<25.0 (*n* = 211)	190 ± 12	123 ± 10	15 ± 3	8 ± 1	16 ± 3	4 ± 1	21 ± 4	4 ± 1
	25.0–29.9 (*n* = 300)	230 ± 11	143 ± 9	8 ± 1	19 ± 4	16 ± 2	3 ± 1	31 ± 4	7 ± 2
	≥30.0 (*n* = 104)	234 ± 18	141 ± 16	4 ± 1	13 ± 3	20 ± 3	5 ± 2	38 ± 7	14 ± 5
	*p–*value	0.03	0.27	<0.01	0.10	0.66	0.81	0.05	0.04
Alcohol consumption	None (*n* = 107)	195 ± 16	107 ± 13	12 ± 3	11 ± 3	21 ± 4	4 ± 1	34 ± 7	6 ± 3
	<1.34 g/day(*n* = 117)	201 ± 16	112 ± 14	13 ± 4	14 ± 3	21 ± 4	7 ± 3	25 ± 6	8 ± 3
	1.34–3.99 g/day (*n* = 126)	217 ± 18	144 ± 15	6 ± 1	18 ± 7	12 ± 2	3 ± 2	24 ± 5	9 ± 4
	4.00–8.93 g/day (*n* = 141)	213 ± 13	149 ± 12	8 ± 1	9 ± 2	13 ± 2	3 ± 1	27 ± 5	4 ± 2
	>8.93 g/day (*n* = 128)	252 ± 18	159 ± 15	10 ± 3	21 ± 7	19 ± 3	3 ± 1	32 ± 6	9 ± 3
	*p–*value	0.11	0.03	0.22	0.40	0.10	0.34	0.70	0.61
Aerobic exercise duration	<101 min/week (*n* = 170)	216 ± 13	132 ± 11	9 ± 2	14 ± 2	21 ± 3	2 ± 1	29 ± 5	9 ± 3
	101–180 min/week (*n* = 151)	210 ± 13	132 ± 12	13 ± 3	11 ± 2	22 ± 4	5 ± 2	25 ± 5	4 ± 2
	181–290 min/week (*n* = 178)	229 ± 15	152 ± 12	9 ± 2	20 ± 7	11 ± 2	4 ± 2	27 ± 5	6 ± 2
	≥291 min/week (*n* = 118)	210 ± 18	124 ± 15	9 ± 2	12 ± 2	14 ± 2	5 ± 2	35 ± 6	12 ± 4
	*p–*value	0.78	0.43	0.55	0.41	0.02	0.50	0.62	0.17
Resistance training duration	<46 min/week (*n* = 191)	238 ± 13	159 ± 12	10 ± 2	13 ± 2	21 ± 3	3 ± 1	23 ± 5	9 ± 3
	46–135 min/week (*n* = 204)	205 ± 11	129 ± 10	10 ± 2	13 ± 2	18 ± 2	5 ± 2	26 ± 4	4 ± 1
	136–270 min/week (*n* = 123)	222 ± 20	137 ± 16	12 ± 3	20 ± 7	13 ± 3	2 ± 1	32 ± 6	7 ± 3
	≥271 min/week (*n* = 95)	192 ± 18	104 ± 11	6 ± 2	16 ± 9	10 ± 2	6 ± 3	40 ± 7	10 ± 4
	*p–*value	0.14	0.03	0.44	0.66	0.04	0.28	0.16	0.46
Sleep duration	≤4 h/night (*n* = 14)	353 ± 93	143 ± 65	7 ± 7	69 ± 59	30 ± 13	3 ± 2	76 ± 29	24 ± 14
	5–6 h/night (*n* = 293)	234 ± 11	148 ± 10	10 ± 2	15 ± 3	19 ± 2	4 ± 1	29 ± 3	10 ± 2
	7–8 h/night (*n* = 287)	197 ± 9	127 ± 8	9 ± 1	12 ± 2	15 ± 2	4 ± 1	25 ± 4	4 ± 1
	≥9 h/night (*n* = 11)	155 ± 30	86 ± 34	20 ± 17	7 ± 3	10 ± 7	9 ± 6	22 ± 11	0 ± 0
	*p–*value	<0.01	0.29	0.53	<0.01	0.32	0.82	0.02	0.04
Rank	Junior enlisted(*n* = 115)	179 ± 17	101 ± 13	10 ± 2	11 ± 2	12 ± 2	6 ± 2	29 ± 6	9 ± 4
	Senior enlisted (*n* = 281)	223 ± 12	129 ± 9	9 ± 1	19 ± 4	16 ± 2	4 ± 1	38 ± 4	8 ± 2
	Warrant officer (*n* = 4)	112 ± 41	50 ± 43	0 ± 0	24 ± 23	24 ± 19	0 ± 0	3 ± 3	11 ± 11
	Junior officer (*n* = 110)	203 ± 15	137 ± 13	4 ± 1	8 ± 2	21 ± 4	1 ± 0	26 ± 6	6 ± 3
	Senior officer (*n* = 109)	257 ± 17	193 ± 16	16 ± 4	13 ± 3	21 ± 4	4 ± 2	6 ± 2	4 ± 2
	*p–*value	<0.01	<0.01	0.06	0.38	0.28	0.28	<0.01	0.71
Special Operations	No (*n* = 592)	217 ± 8	138 ± 6	9 ± 1	15 ± 2	17 ± 1	4 ± 1	28 ± 3	7 ± 1
	Yes (*n* = 24)	187 ± 22	98 ± 24	4 ± 2	11 ± 4	15 ± 7	0 ± 0	52 ± 15	6 ± 6
	*p-*value	0.42	0.22	0.32	0.73	0.77	0.32	0.06	0.92

Abbreviation: SE = standard error.

**Table 7:** Characteristics associated with use (≥1 time/week) of specific caffeine products among Navy personnel. Multivariable logistic regression was used. Data are presented as odds ratios adjusted for other factors listed with 95% confidence intervals. The odd ratio 1.00 represents the reference group for each variable.

Variable	Strata	Caffeine Beverage or Gum/Medication Consumed ≥1 Time/Week					
		Any Caffeine	Coffee	Tea *	Soda ^†^	Energy Drink	Gum/Medication
		(Model 1)	(Model 2)	(Model 3)	(Model 4)	(Model 5)	(Model 6)
Gender	Men	1.00	1.00	1.00	1.00	1.00	1.00
	Women	0.69 (0.35–1.38)	0.55 (0.35–0.86)	1.56 (1.04–2.34)	0.61 (0.41–0.91)	0.43 (0.25–0.75)	2.57 (1.31–5.06)
Age (years)	18–24	1.00	1.00	1.00	1.00	1.00	1.00
	25–29	2.00 (0.82–4.87)	1.27 (0.72–2.24)	1.07 (0.63–1.83)	1.29 (0.77–2.19)	1.58 (0.84–2.96)	0.82 (0.33–2.06)
	30–39	2.23 (0.91–5.04)	1.77 (0.98–3.18)	1.14 (0.66–1.98)	1.15 (0.67–1.96)	0.86 (0.43–1.67)	1.12 (0.45–2.79)
	≥40	3.23 (1.01–5.45)	1.52 (0.78–2.96)	1.25 (0.67–2.32)	1.03 (0.56–1.89)	0.31 (0.18–0.98)	1.91 (0.71–5.14)
Education	Some high school/high school graduate	1.00	1.00	1.00	1.00	1.00	1.00
	Some college	1.40 (0.63–3.07)	1.65 (0.96–2.84)	0.88 (0.53–1.48)	1.58 (0.95–2.64)	1.49 (0.80–2.78)	0.83 (0.36–1.88)
	College degree	1.10 (0.42–2.46)	1.79 (1.02–2.54)	0.71 (0.41–1.25)	1.36 (0.78–2.36)	0.78 (0.39–1.57)	0.57 (0.22–1.43)
Marital status	Single	1.00	1.00	1.00	1.00	1.00	1.00
	Married	1.11 (0.59–2.07)	1.13 (0.75–1.70)	1.25 (0.86–1.83)	1.13 (0.78–1.63)	0.54 (0.34–0.84)	0.90 (0.48–1.68)
Race/ethnicity	White	1.00	1.00	1.00	1.00	1.00	1.00
	Black	0.14 (0.07–0.28)	0.24 (0.14–0.40)	0.73 (0.44–1.21)	0.63 (0.39–1.03)	0.34 (0.17–0.71)	0.68 (0.30–1.53)
	Hispanic	0.66 (0.27–1.60)	1.13 (0.60–2.13)	1.02 (0.58–1.80)	0.82 (0.47–1.44)	0.96 (0.48–1.89)	0.42 (0.12–1.46)
	Other	0.90 (0.29–2.80)	0.87 (0.46–1.67)	1.24 (0.71–2.20)	0.73 (0.41–1.29)	0.78 (0.36–1.68)	0.58 (0.19–1.74)
Body mass index (kg/m^2^)	<25.0	1.00	1.00	1.00	1.00	1.00	1.00
	25.0–29.9	1.07 (0.57–2.00)	1.41 (0.94–2.13)	1.00 (0.69–1.45)	1.05 (0.73–1.51)	1.79 (1.11–2.91)	1.19 (0.62–2.29)
	≥30.0	1.93 (0.75–4.93)	1.23 (0.71–2.16)	0.85 (0.50–1.44)	1.02 (0.61–1.68)	2.21 (1.16–4.23)	1.84 (0.80–4.23)
Alcohol consumption	None	1.00	1.00	1.00	1.00	1.00	1.00
	<1.33 g/day	2.32 (1.08–4.97)	1.08 (0.63–1.86)	0.96 (0.56–1.64)	1.11 (0.67–1.86)	1.53 (0.77–3.05)	1.49 (0.58–3.82)
	1.33–3.99 g/day	4.38 (1.82–10.56)	1.86 (1.06–3.25)	1.23 (0.72–2.10)	0.93 (0.56–1.56)	1.09 (0.54–2.18)	2.00 (0.79–5.07)
	4.00–8.91 g/day	2.96 (1.28–6.86)	2.49 (1.40–4.43)	1.57 (0.93–2.65)	0.83 (0.50–1.37)	1.58 (0.81–3.09)	0.82 (0.28–2.36)
	≥8.92 g/day	6.66 (2.35–18.90)	2.66 (1.49–4.75)	1.24 (0.73–2.11)	1.24 (0.74–2.08)	2.01 (1.05–3.86)	2.68 (1.11–6.52)
Aerobic exercise duration	<101 min/week	0.63 (0.26–1.49)	0.70 (0.40–1.24)	0.91 (0.55–1.52)	1.19 (0.72–1.96)	0.89 (0.48–1.62)	0.82 (0.37–1.81)
	101–180 min/week	0.66 (0.26–1.69)	0.60 (0.33–1.07)	0.61 (0.36–1.05)	0.96 (0.57–1.60)	0.67 (0.35–1.30)	0.43 (0.18–1.05)
	181–290 min/week	0.74 (0.31–1.78)	0.88 (0.50–1.54)	0.91 (0.55–1.50)	1.00 (0.61–1.63)	0.63 (0.34–1.16)	0.55 (0.24–1.23)
	≥291 min/week	1.00	1.00	1.00	1.00	1.00	1.00
Resistance training duration	<46 min/week	1.99 (0.80–5.00)	1.31 (0.72–2.39)	1.03 (0.59–1.80)	1.36 (0.79–2.35)	0.63 (0.32–1.23)	1.10 (0.43–2.77)
	46–135 min/week	1.80 (0.76–4.28)	1.52 (0.85–2.72)	1.26 (0.74–2.16)	1.57 (0.93–2.65)	1.15 (0.61–2.14)	1.06 (0.43–2.60)
	136–270 min/week	1.45 (0.26–3.60)	1.28 (0.69–2.36)	1.07 (0.60–1.90)	1.00 (0.58–1.76)	1.08 (0.55–2.11)	0.89 (0.34–2.33)
	≥271 min/week	1.00	1.00	1.00	1.00	1.00	1.00
Sleep duration	≤4 h/night	2.35 (0.26–21.56)	0.45 (0.14–1.50)	0.74 (0.22–2.50)	0.98 (0.31–3.07)	2.20 (0.66–7.33)	2.53 (0.57–11.31)
	5–6 h/night	1.68 (0.94–2.99)	1.37 (0.94–1.99)	0.97 (0.69–1.35)	1.32 (0.95–1.82)	1.31 (0.86–1.99)	1.40 (0.79–2.49)
	7–8 h/night	1.00	1.00	1.00	1.00	1.00	1.00
	≥9 h/night	2.24 (0.24–20.98)	0.95 (0.27–3.40)	0.88 (0.25–3.09)	0.58 (0.16–2.08)	2.02 (0.54–7.64)	0.94 (0.10–8.61)
Nagelkerke *R*^2^		0.23	0.18	0.04	0.07	0.18	0.11

* Includes hot and other teas. ^†^ Includes cola-type beverages and other sodas.

**Table 8:** Characteristics associated with use (≥1 time/week) of specific caffeine products among Marine Corps personnel. Multivariable logistic regression was used. Data are presented as odds ratios adjusted for other factors listed with 95% confidence intervals. The odds ratio 1.00 represents the reference group for each variable.

Variable	Strata	Caffeine Beverage or Gum/Medication Consumed ≥1 Time/Week					
		Any Caffeine	Coffee	Tea *	Soda ^†^	Energy Drink	Gum/Medication
		(Model 1)	(Model 2)	(Model 3)	(Model 4)	(Model 5)	(Model 6)
Gender	Men	1.00	1.00	1.00	1.00	1.00	1.00
	Women	1.08 (0.66–1.77)	1.17 (0.82–1.68)	1.45 (1.02–2.07)	0.58 (0.41–0.82)	0.64 (0.43–0.93)	3.15 (1.66–5.99)
Age (years)	18–24	1.00	1.00	1.00	1.00	1.00	1.00
	25–29	1.93 (1.11–3.38)	1.11 (0.74–1.67)	0.93 (0.62–1.41)	0.98 (0.65–1.47)	1.13 (0.74–1.72)	0.49 (0.22–1.09)
	30–39	3.59 (1.85–6.96)	1.76 (1.13–2.73)	0.91 (0.59–1.40)	1.81 (1.19–2.74)	0.93 (0.60–1.44)	0.77 (0.37–1.62)
	≥40	3.15 (1.22–8.11)	1.61 (0.88–2.93)	0.98 (0.55–1.75)	1.51 (0.87–2.62)	0.32 (0.17–0.62)	1.60 (0.62–4.13)
Education	Some high school/high school graduate	1.00	1.00	1.00	1.00	1.00	1.00
	Some college	0.97 (0.61–1.55)	1.16 (0.82–1.65)	1.01 (0.71–1.45)	0.65 (0.46–0.92)	1.29 (0.90–1.87)	1.65 (0.87–3.15)
	College degree	1.39 (0.66–2.92)	1.83 (1.14–2.94)	0.93 (0.59–1.47)	0.64 (0.41–0.99)	0.58 (0.36–0.94)	0.88 (0.36–2.16)
Marital status	Single	1.00	1.00	1.00	1.00	1.00	1.00
	Married	1.32 (0.84–2.07)	1.22 (0.87–1.71)	0.86 (0.61–1.20)	1.22 (0.88–1.70)	1.24 (0.87–1.77)	1.56 (0.83–2.92)
Race/ethnicity	White	1.00	1.00	1.00	1.00	1.00	1.00
	Black	0.59 (0.35–0.96)	0.44 (0.27–0.73)	1.27 (0.77–2.08)	0.74 (0.44–1.21)	0.54 (0.31–0.89)	1.05 (0.48–2.29)
	Hispanic	0.77 (0.37–1.58)	0.95 (0.65–1.38)	0.87 (0.60–1.27)	0.54 (0.38–0.78)	0.46 (0.31–0.70)	0.89 (0.47–1.68)
	Other	0.40 (0.22–0.75)	0.56 (0.34–0.93)	1.44 (0.88–2.35)	0.76 (0.47–1.24)	0.56 (0.32–0.96)	0.41 (0.13–1.24)
Body mass index (kg/m^2^)	<25.0	1.00	1.00	1.00	1.00	1.00	1.00
	25.0–29.9	0.77 (0.49–1.23)	1.40 (1.02–1.95)	0.86 (0.62–1.19)	0.81 (0.59–1.11)	1.21 (0.87–1.70)	1.64 (0.89–3.02)
	≥30.0	1.04 (0.35–3.04)	1.83 (0.88–3.81)	0.82 (0.41–1.67)	0.61 (0.31–1.18)	1.31 (0.66–2.59)	3.48 (1.24–9.74)
Alcohol consumption	None	1.00	1.00	1.00	1.00	1.00	1.00
	<1.33 g/day	1.57 (0.89–2.76)	1.87 (1.23–2.86)	1.42 (0.92–2.18)	1.08 (0.71–1.64)	1.95 (1.23–3.10)	1.21 (0.58–2.49)
	1.33–3.99 g/day	2.13 (1.14–3.98)	2.48 (1.59–3.84)	1.15 (0.73–1.80)	1.30 (0.85–1.98)	1.65 (1.03–2.63)	0.84 (0.38–1.84)
	4.00–8.91 g/day	2.25 (1.16–4.37)	2.75 (1.75–4.35)	1.49 (0.95–2.34)	1.45 (0.74–1.77)	1.65 (1.02–2.67)	1.07 (0.48–2.36)
	≥8.92 g/day	2.27 (1.18–4.39)	3.37 (2.12–5.36)	1.45 (0.92–2.27)	1.44 (0.94–2.21)	1.82 (1.13–2.92)	1.05 (0.46–2.36)
Aerobic exercise duration	<101 min/week	0.92 (0.50–1.68)	0.81 (0.52–1.26)	1.01 (0.65–1.55)	1.10 (0.73–1.67)	1.09 (0.70–1.72)	0.35 (0.15–0.83)
	101–180 min/week	1.19 (0.64–2.19)	0.90 (0.58–1.40)	1.01 (0.65–1.55)	1.42 (0.94–2.15)	1.17 (0.75–1.83)	0.63 (0.29–1.37)
	181–290 min/week	0.99 (0.56–1.79)	0.83 (0.54–1.26)	1.20 (0.80–1.80)	1.07 (0.72–1.58)	1.26 (0.82–1.93)	0.93 (0.48–1.81)
	≥291 min/week	1.00	1.00	1.00	1.00	1.00	1.00
Resistance training duration	<46 min/week	1.35 (0.70–2.60)	1.02 (0.64–1.62)	1.09 (0.70–1.71)	1.81 (1.15–2.82)	0.87 (0.54–1.39)	0.84 (0.38–1.86)
	46–135 min/week	1.56 (0.83–2.91)	1.39 (0.89–2.16)	0.76 (0.50–1.18)	1.42 (0.94–2.15)	0.98 (0.63–1.52)	0.53 (0.24–1.16)
	136–270 min/week	0.98 (0.56–1.71)	0.88 (0.58–1.35)	0.74 (0.49–1.12)	1.17 (0.78–1.74)	0.87 (0.57–1.34)	0.83 (0.40–1.69)
	≥271 min/week	1.00	1.00	1.00	1.00	1.00	1.00
Sleep duration	≤4 h/night	0.85 (0.37–1.96)	0.82 (0.42–1.58)	1.15 (0.60–2.21)	1.80 (0.94–3.43)	4.17 (2.14–8.14)	4.16 (1.51–11.47)
	5–6 h/night	1.36 (0.88–2.11)	1.16 (0.85–1.59)	0.91 (0.68–1.23)	1.22 (0.91–1.63)	2.17 (1.57–3.00)	2.38 (1.30–4.36)
	7–8 h/night	1.00	1.00	1.00	1.00	1.00	1.00
	≥9 h/night	0.41 (0.13–1.29)	0.92 (0.30–2.77)	0.60 (0.18–1.94)	0.53 (0.16–1.72)	0.54 (0.11–2.53)	2.67 (0.52–13.96)
Nagelkerke *R*^2^		0.17	0.16	0.04	0.11	0.16	0.13

* Includes hot and other teas. ^†^ Includes cola-type beverages and other sodas.

## Disclaimers

The views expressed in this research are those of the authors and do not necessarily reflect the official policy or position of the Department of the Navy, Department of the Army, Department of the Air Force, Department of Veterans Affairs, Department of Defense, or the US Government. Approved for public release; distribution is unlimited. US Government Work (17 USC §105). Not copyrighted in the United States. Human subjects participated in this study after giving their free and informed consent. This research has been conducted in compliance with all applicable federal regulations governing the protection of human subjects in research (Protocol NHRC.2013.0009).
